# Supplementary material for: Reconciling Mining with the Conservation of Cave Biodiversity: A Quantitative Baseline to Help Establish Conservation Priorities
Source: PLoS One. 2016 Dec 20;11(12):e0168348. doi: 10.1371/journal.pone.0168348 (PMC5173368; doi:10.1371/journal.pone.0168348)
Supplement: S1 Dataset — (ZIP) [file pone.0168348.s002.zip › Taxa/Serra Norte/SN_2013/N4E/Lista N4E-69.pdf]

CAVIDADE N4E-0069

Classe	Ordem	Fam/Outros	Gên/Outros	Espécie	S-U	U-S	Zon.
Annelida	Oligochaeta			jovem		1	P
Arachnida	Araneae	Corinnidae		jovem	1		P
Arachnida	Araneae	Ctenidae		jovem	3	1	P
Arachnida	Araneae	Pholcidae	*Mesabolivar*	*aurantiacus*	X	X	E/P
Arachnida	Araneae	Pholcidae	*Mesabolivar*	sp.1	X		E/P
Arachnida	Araneae	Pisauridae	*Thaumasia*	*velox*	2	1	E/P
Arachnida	Araneae	Theridiidae	*Dipoena*	sp.5	X		P
Arachnida	Araneae	Theridiosomatidae	*Plato*	sp.1	X	X	P
Arachnida	Araneae	Zodariidae		jovem	2		P
Arachnida	Opiliones	Sclerosomatidae	*Prionostema*	sp.1	7	1	E/P
Arachnida	Opiliones	Stygnidae	*Protimesius*	*gracilis*	1		E
Diplopoda	Polydesmida	Chelodesmidae		sp.3		1	P
Diplopoda	Spirostreptida	Pseudonannolenidae	*Pseudonannolene*	sp.2		1	E
Entognatha	Collembola	Paronellidae		sp.1	X		P
Insecta	Archaeognatha	Meinertellidae		sp.1		X	E
Insecta	Blattodea	Blattidae		sp.2	4		E
Insecta	Coleoptera	Staphylinidae	Pselaphinae	sp.36	X		P
Insecta	Coleoptera	Staphylinidae		sp.40	X		P
Insecta	Coleoptera	Staphylinidae		sp.43	X		P
Insecta	Coleoptera			jovem		X	P
Insecta	Diptera	Culicidae	*Culex*	sp.	X		P
Insecta	Diptera	Limoniidae		sp.	X		E/P
Insecta	Diptera	Tipulidae	Tipulinae	sp.	X	X	E/P
Insecta	Heteroptera	Gerridae	*Tachygerris*	jovem	X		P
Insecta	Heteroptera	Hebridae		sp.	X		P
Insecta	Heteroptera	Hydrometridae		sp.	X		P
Insecta	Heteroptera	Veliidae	*Microvelia*	sp.	X		P
Insecta	Heteroptera	Veliidae	*Paravelia*	sp.	X	X	P
Insecta	Heteroptera	Veliidae	*Rhagovelia*	jovem	X	X	P
Insecta	Hymenoptera	Diapriidae		sp.2	X		E
Insecta	Hymenoptera	Formicidae	*Camponotus*	sp.2	X		P
Insecta	Hymenoptera	Formicidae	*Crematogaster*	sp.1	X		P
Insecta	Hymenoptera	Formicidae	Myrmicinae	sp.1	X		P
Insecta	Hymenoptera	Formicidae	*Odontomachus*	sp.1	X		P
Insecta	Hymenoptera	Formicidae	*Pheidole*	sp.1	X		P
Insecta	Isoptera	Termitidae	*Nasutitermes*	sp.	X		P
Insecta	Odonata	Corduliidae		sp.	1	2	E/P
Insecta	Orthoptera	Phalangopsidae	*Phalangopsis*	jovem	2		P
Amphibia	Anura	Strabomantidae	*Pristimantis*	cf. *fenestratus*	14	3	E/P
Mammalia	Chiroptera	Emballonuridae	*Peropteryx*	*kappleri*	1	2	P
